# Left Ventricular Rigid Body Rotation in Ebstein's Anomaly from the MAGYAR-Path Study

**DOI:** 10.5935/abc.20160050

**Published:** 2016-06

**Authors:** Attila Nemes, Kálmán Havasi, Péter Domsik, Anita Kalapos, Tamás Forster

**Affiliations:** 2nd Department of Medicine and Cardiology Center, University of Szeged, Szeged - Hungary

**Figure 1:**
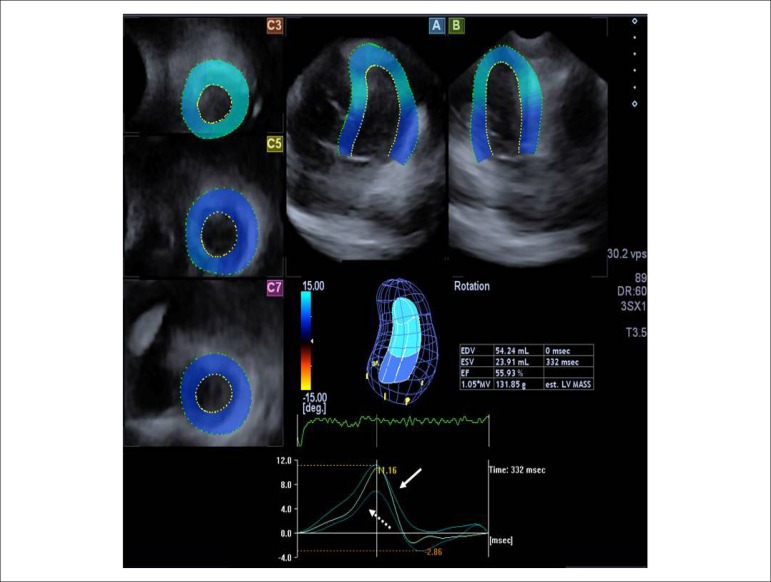
Apical 4-chamber (A) and 2-chamber (B) views and short–axis views (C3, C5, C7) at different levels of the left ventricle (LV) extracted from the three-dimensional (3D) echocardiographic dataset are shown in the patient with Ebstein’s anomaly. The 3D image of the LV and calculated LV volumetric and functional characteristics (EDV: end-diastolic volume; ESV: end-systolic volume; EF: ejection fraction) are also demonstrated together with LV apical (white arrow), mid-ventricular and basal (dashed arrow) rotations in the same counterclockwise direction, confirming absence of the LV twist, called “rigid body rotation”.

A 70-year-old female patient with Ebstein's anomaly (EA) that had never undergone palliation was assessed (the case originates from the MAGYAR-Path Study). Complete two-dimensional (2D) Doppler and three-dimensional (3D) speckle-tracking echocardiography were carried out with commercially available Toshiba Artida^™^ echocardiography equipment. During 2D echocardiography, the septal leaflet-tricuspid annulus distance showed to be 25 mm, confirming EA. While the right ventricle (RV) was enlarged with tricuspid annular plane systolic excursion > 23 mm and mitral regurgitation grade III, left ventricular (LV) size and function showed to be normal with an ejection fraction of 56% without wall motion abnormalities. However, all LV regions moved in almost the same counterclockwise direction, confirming absence of LV twist, called "rigid body rotation" (RBR) (Figure 1). The mean global LV radial, circumferential, longitudinal, 3D and area strain parameters showed to be 11.5 ± 10.0%, -25.5 ± 15.4%, -18.6 ± 10.2%, 15.2 ± 10.8% and -34.7 ± 20.8%, respectively. EA is a congenital heart defect in which septal and posterior leaflets of the tricuspid valve are displaced towards the RV apex, leading to RV partial atrialization, although the anatomic annulus of the valve is in the normal position.^1^ Malformation and displacement of the anterior leaflet can also be present. To the best of our knowledge, this is the first report to demonstrate LV-RBR, a known feature in LV myocardial mechanics, in a single patient with unrepaired EA. LV-RBR could be partially explained by the impaired ventricle-to-ventricle interactions due to displaced tricuspid valve leaflet attachments, alterations in the anatomic myocardial fiber orientation, but other reasons could also not be excluded.
